# The role of childhood traumas on father-child sexual communication language: Self-esteem, social anxiety and sexual education

**DOI:** 10.1371/journal.pone.0340776

**Published:** 2026-03-05

**Authors:** Ibrahim Güngör, Ismihan Zeliha Artan

**Affiliations:** 1 Department of Child Development, Faculty of Health Sciences, Bingöl University, Bingöl, Turkey; 2 Department of Child Development, Faculty of Health Sciences, Hacettepe University, Ankara, Turkey; Karabük Üniversitesi: Karabuk Universitesi, TÜRKIYE

## Abstract

The aim of the study was to examine the direct and indirect associations between fathers’ childhood abuse experiences and their levels of self-esteem, social anxiety, attitudes towards sexual education and sexual communication language levels with their children. The sample of the study consisted of 587 fathers. Data were collected through ‘Childhood Trauma Questionnaire Short Form’, ‘Social Anxiety Scale Short Form’, ‘Rosenberg Self-Esteem Scale’, ‘Attitude Towards Sexual Education Scale’, ‘Sexual Communication Scale for Parents’ and ‘Demographic Information Form’. The data were analyzed using Pearson correlation coefficients and path analysis conducted in SPSS 24 and R. The findings showed that childhood abuse experiences were positively correlated with social anxiety and negatively correlated with self-esteem and father’s sexual communication language with his child. As a result of the path analysis, it was found that childhood abuse experiences were positively associated with social anxiety and negatively associated with self-esteem and sexual communication language. Self-esteem was negatively associated with social anxiety and positively associated with sexual communication language; social anxiety was negatively associated with attitudes towards sexual education and sexual communication language; and attitudes towards sexual education were positively associated with sexual communication language. Childhood abuse experiences were also indirectly associated with social anxiety, attitudes towards sexual education, and sexual communication language through self-esteem and social anxiety. Overall, the findings were discussed within the framework of father-child sexual communication language and fathers’ involvement in the sexual education process.

## Introduction

Sexual development encompasses not only the development and growth of reproductive organs but also the acquisition of sexual knowledge, beliefs and learned behaviors. Broadly defined, sexuality includes biological, physiological, psychological, social, cultural, behavioral, moral and educational issues related to all aspects of being a woman and/or a man [1]. Sexuality is a lifelong learning process that begins within the family and continues through teachers, peer groups, physicians, health professionals and the media. Everyone has individual, familial and social responsibilities regarding their own sexuality and the sexuality of the people around them. Sexual education and the development of a sexual communication language are particularly important for children, as they facilitate a healthy transition into adulthood [2,3]. Sexuality significantly influences an individual’s happiness, success, and interpersonal relationships. Therefore, the necessity of sexual education and the importance of sexual communication are indisputable [1]. However, single-cause and one-dimensional explanations of human behavior are often insufficient, because the factors influencing individuals’ behaviors are interconnected and intertwined [4–6].

Parenting is one of the most important factors affecting child development and can shape its course. Parents provide various care activities and support to their children in different contexts to promote their well-being. The more effectively parents fulfill parenting responsibilities, the more positively their children’s life adaptation, mental health, and well-being in adulthood are affected [7,8]. Discussions on how the differences and similarities of motherhood and fatherhood affect children’s development continue across cultures, beliefs, biological conditions, social and economic changes [7,9–12]. However, when children have emotional, economic, and functional access to parents, children’s development benefits the most from the complementary characteristics of parents [7,9,10]. At this point, it can be said that it is important for both mothers and the fathers to participate in the child’s development and life as much as possible. Like all developmental areas, sexual development starts in the womb and continues throughout life. However, compared to other areas of development, issues related to sexuality and sexual development are generally considered taboo and are often avoided [13,14].

Although parents are aware of the importance of their role in sexual education, many report that they are not sufficiently prepared to communicate with their children about sexuality, lacked adequate information, and feared disrupting their children’s innocence [6,14–18]. It has been reported that mothers take a greater role in sexual education and communication than fathers [19–22], and also that fathers often perceive child sexual education as part of the mothers’ responsibilities due to the prevailing cultural norms regarding motherhood and fatherhood [23–25]. Although there is a growing body of literature on the difficulties fathers face in taking on this role, there are limited studies specifically fathers’ experiences with sexual communication before and after puberty, as well as the reasons they report such difficulties. While mothers often take on the role of emotional nurturers and primary educators in children’s early development, fathers tend to influence children’s understanding of autonomy, boundary-setting, and social behavior through more task-oriented and modeling-based interactions. In the context of sexual education, this difference means that fathers are more likely to shape how children perceive masculinity, respect, and protective behavior in relationships, complementing the emotional and informational guidance typically provided by mothers [19–22,26–28]. However, studies have shown that the amount of time fathers spend with their children positively affects children’s overall development, including sexual development, depending on the quality of communication and the parent-child relationship. Attachment and social learning theories suggest that paternal communication, particularly regarding sensitive topics such as sexuality, provides unique modeling opportunities that differ from maternal interactions. Fathers’ engagement in open and supportive discussions can foster children’s confidence and reduce the secrecy or anxiety often surrounding sexual topics. Conversely, limited paternal involvement driven by cultural taboos, masculinity expectations, or discomfort may perpetuate silence and misinformation [6,26–28]. In the light of all this information, it is highly important to understand the factors affecting fathers’ sexual communication process with their children.

The aim of this study is to examine the relationship between fathers’ childhood abuse, self-esteem, social anxiety and attitudes towards sexual education as well as their sexual communication language with their children. In this way, it was aimed to determine the predictors influencing fathers’ attitudes towards sexual education and their sexual communication with their children. Within the framework of this purpose, a model was created to positively enhance fathers’ attitudes towards sexual education and sexual communication and to ensure that fathers participate as fully as possible in their children’s sexual development and overall lives. This study is important for understanding the factors that underlying fathers’ attitudes toward sexual education and the language they use when communicating with their children about sexual matters. It differs from previous research by focusing specifically on fathers and by integrating multiple psychological variables such as childhood trauma, self-esteem, social anxiety, and attitudes toward sex education into a single structural model. In many cultural contexts, including Turkey, discussions of sexuality are often influenced by traditional gender norms and taboos. Fathers are typically socialized to be more distant or reserved in conversations about emotional and sexual matters, which has historically limited their involvement in their children’s sexual education. However, changing family dynamics and evolving concepts of fatherhood have underscored the importance of fathers as active participants in promoting children’s healthy sexual development. Focusing specifically on fathers, rather than parents more broadly, allows for an in-depth understanding of how masculinity norms, cultural expectations, and social roles influence fathers’ comfort, openness, and responsibility in sexual communication with their children. It also addresses a significant gap in the existing literature within culturally conservative contexts such as Turkey.

### Theoretical framework

It is stated that anxiety and self-esteem originate from childhood experiences [29]. Studies have shown that childhood abuse experiences increase anxiety [30–33] and lower self-esteem [34–37]. Similarly, childhood abuse experiences are reported to negatively affect close relationships [38,39]. Self-esteem also shows a negative relationship with anxiety [30,31]. It is known that in anxious situations, individuals can react to new circumstances and responsibilities with either struggle or avoidance. The severity and intensity of the situation determine this process. Environmental factors, as well as patterns of feeling, thinking, and behavior, can cause the anxiety process to persist in a cyclical manner [29]. Indeed, parents’ experiences of abuse and neglect in their own childhood can even affect the mental processes of their children [40]. Similarly, it is stated that parents’ experiences of sexual education received from their own parents influence their communication with their children [41]. Previous research suggests that the intergenerational effects of childhood trauma may operate through internalized psychological mechanisms such as anxiety and self-esteem. Fathers who experienced abuse or neglect in their own childhood may develop increased anxiety or reduced self-worth, which can influence their emotional availability and comfort in communicating about sexuality with their children. Anxiety may lead to avoidance or tension during discussions about sensitive topics, whereas low self-esteem can undermine confidence in one’s parental role or educational abilities. In this respect, anxiety and self-esteem serve as potential mediators that transmit the long-term effects of early trauma to fathers’ sexual communication language and educational attitudes, thereby providing a coherent explanatory pathway within the conceptual model [42–44].

In this study, the concept of “sexual communication language” refers to the verbal and non-verbal expressions used by parents particularly fathers when discussing sexuality with their children. This construct encompasses several dimensions, including the comfort level, openness, content, tone, and emotional quality of communication. Rather than representing only the amount of information shared, it reflects the overall communicative climate, indicating how effectively and comfortably parents convey sexual knowledge, values, and attitudes. In this sense, sexual communication language captures both the informational and affective components of parent–child dialogue about sexuality [45]. Studies also indicate that parents’ comfort level in discussing sexuality affects their communication with their children on this topic [6,17,18].

### Methodological framework

In this study, Structural Equation Modelling (SEM) was used as the main methodology to examine the directional associations among various psychological constructs. The dataset was loaded and preprocessed using the R programming language, with all variables converted to numerical values to facilitate statistical analysis. The SEM method was preferred because of its robustness in handling latent variables and its ability to model complex interrelationships between observed and unobserved variables. Before estimating the structural model, Confirmatory Factor Analyses (CFAs) were conducted for each latent construct to confirm measurement validity. This step ensured that all latent variables such as childhood trauma, self-esteem, social anxiety, and attitudes toward sexual education were reliably represented by their observed indicators. Only after achieving satisfactory measurement model fit were the structural paths among these constructs estimated. Model refinement studies began with the initial model, which incorporated theoretically hypothesized directional associations between childhood trauma and outcomes such as self-esteem, social anxiety, attitudes towards sexual education, and father-child communication, with multiple iterations performed. Indirect pathways were also hypothesized to assess the mediating roles of self-esteem, social anxiety and attitudes toward sex education.

Throughout the refinement process, non-significant paths were evaluated carefully: paths were removed only if they lacked theoretical justification in prior literature. Conversely, when modification indices suggested adding covariances between certain error terms, these adjustments were applied only when a meaningful conceptual rationale could be established (e.g., overlapping content or measurement similarity) in line with prior evidence suggesting conceptual overlap among indicators of emotional distress and self-related constructs in trauma-exposed populations [43,46]. This dual approach balancing empirical model improvement with theoretical grounding ensured that the model refinement process did not rely solely on statistical optimization but reflected sound conceptual reasoning. The models were tested using maximum likelihood estimation and fit indices were calculated to assess validity; this process led to further refinements based on modification indices and theoretical justifications [47,48]. Throughout the model refinement process, alternative models were compared using changes in χ²/df ratios, CFI, and RMSEA values to ensure parsimony and theoretical consistency.

The refined SEM model emphasized the importance of childhood trauma in predicting various psychological and social outcomes by incorporating multiple measurement models and structural pathways. Structural paths examined the relationships between childhood trauma and the outcomes, considering self-esteem, social anxiety, and attitudes toward sex education as mediating variables. This methodological framework not only enabled an in-depth exploration of theoretical constructs but also provided actionable insights into the dynamics of the long-term effects of childhood trauma, which aligns with previous findings highlighting the mediating roles of self-esteem and social anxiety in the relationship between early adversity and interpersonal functioning [42,49], as evidenced by the statistical outputs and refined pathways identified in the final SEM model.

Comprehensive details of the measurement validation and model refinement process including CFA results, item retention decisions, structural path adjustments, and theoretical rationales are presented in S1-S5 Tables.

## Method

### Participants and procedure

The population of our cross-sectional study consisted of children aged 3–11 years and their fathers. Since many factors beyond biological and familial influences such as peer relationships cultural values, digital media exposure, and individual experiences affect a child’s sexual development as they grow, the sample was limited to the childhood period. The lower age limit was set at 3 years because this is a critical period during which children begin to develop their sexual identities, their sexual curiosity increases, and they acquire sexual roles by identifying with their own sex [50]. The upper age limit was set at 11 years due to the rapid development of physical and behavioural changes, the emergence of secondary sex characteristics indicating the onset of reproductive maturity and the beginning of puberty [51]. Although the 3–11 age range covers distinct developmental stages, this period constitutes a continuous developmental process in which children’s understanding of sexuality, curiosity, and communication patterns gradually evolve within the broader framework of early social learning. Since the present study focused on fathers’ language and attitudes when discussing sexual issues, rather than on children’s developmental outcomes, children across all stages of childhood were included in the sample.

This study included fathers who met the following criteria: having at least one child between 3 and 11 years of age, living in the same household with their child(ren), voluntarily agreeing to participate, being able to read and understand Turkish, and maintaining regular contact with at least one of their children. Fathers who withdrew from the study at any stage of the data collection process were excluded. Since the study was conducted in school settings and based on self-reported data, no psychiatric examination or medical record review was performed. Fathers were not clinically screened for psychiatric disorders or medication use.

The minimum sample size was determined as 400 at 5% precision and 95% confidence interval [52]. Additionally, in our study, the achieved sample size was consistent with simulation-based recommendations for mediation and structural equation modeling (SEM) analyses. According to Fritz and MacKinnon [53], detecting small-to-medium indirect effects with 0.80 power requires approximately 400–500 participants, indicating that our sample size met these statistical power requirements [53].

Considering the possibility of missing data, we collected the data in a manner that ensured the minimum sample size was maintained. Before starting the study, we obtained approval from Hacettepe University Ethics Commission (E-66777842-300-00003429066) and the necessary permissions were obtained from Ankara Provincial Directorate of National Education on 15.03.2024 with the number (E-14588481-605.99-99470576). We complied with the ethical standards specified in the 1964 Helsinki Declaration at every stage of our research. The data collection process began between 22 March 2024 and 16 June 2024 by first meeting with school administrators in the Altındağ, Çankaya, Mamak, and Yenimahalle districts of Ankara and collecting the forms from sealed envelopes. However, many fathers stated that they did not want to fill out the forms by hand, fearing that their responses might be seen by their children or others. As in many societies, talking about sexuality is considered a taboo in Turkey [13,14]. To make the fathers feel more comfortable, an online Google Form containing information about the research and a consent form was created and sent to the fathers through the school administrations and data were subsequently collected. After the Google Form was created, the study was also announced on WhatsApp and Instagram, two social media platforms. The link to the Google Form was sent to the fathers who saw the announcement and wished to participate. Only participants who voluntarily agreed to participate after being informed were included in the study. Participants were asked not to share personal information to ensure confidentiality and anonymity. Flexibility was also provided, allowing participants to discontinue the study at any stage of the form if they wished.

A purposive sampling method was employed to select the study participants. Initially, potential participants were identified through cooperation with school administrations in the Altındağ, Çankaya, Mamak, and Yenimahalle districts of Ankara. Fathers of children aged 3–11 years who were enrolled in these schools were invited to participate in the study. Later, to increase diversity and accessibility, the same questionnaire was distributed via online platforms such as WhatsApp and Instagram. Participation was entirely voluntary, and only fathers who met the inclusion criteria and consented to participate were included in the study. The purposive sampling approach was preferred because the study aimed to include only fathers who shared the same household with their children and were actively involved in their daily communication and upbringing, even if for limited periods during the day. Fathers with more than one child were asked to respond based on only one of their children aged 3–11 years. In total, data were collected from 614 fathers, but the data of 27 fathers who met the exclusion criteria were excluded from the analyses, of whom 19 did not live in the same household with their children and 8 reported that they did not spend time with their children during the day. The demographic characteristics of the final sample are presented in Table 1.

**Table 1:** Distribution of demographic characteristics of the fathers and children.

	Variables	Groups	F	%
Father	Age	25-35	156	26.6
		36-45	356	60.6
		46 +	75	12.8
	Education Level	Primary education	52	8.9
		High school	151	25.7
		Associate degree	48	8.2
		Bachelor’s degree	258	44.0
		Postgraduate	78	13.3
	Sense of involvement in the child’s development and life	Sometimes	151	25.7
		Mostly	316	53.8
		Always	120	20.4
	Time spent with the child during the day	Less than 1 hour	48	8.2
		1-3 hours	309	52.6
		4 hours or more	230	39.2
	Feeling competent in answering questions about the child’s sexual development	Not competent	55	9.4
		Very little competent	199	33.9
		Competent	264	45.0
		Very competent	69	11.8
	Seeking information about children’s sexual development	Yes	182	31.0
		No	405	69.0
Child	Gender	Female	271	46.2
		Male	316	53.8
	Age	3-6	257	43.8
		7-11	330	56.2
	Number of children	Single child	188	32.0
		2 children	300	51.1
		3 or more children	99	16.9
	Gender distribution of all children that fathers have	Only daughters	161	27.4
		Only sons	206	35.1
		Both sons and daughters	220	37.5
Family	Household income (Turkish lira [TL])	0-20.000 TL	69	11.8
		20.001-40.000 TL	162	27.6
		40.001-60.000 TL	136	23.2
		60.001-80.000 TL	83	14.1
		80.001 TL and above	137	23.3
		Total	587	100

When the table is analysed, 26.6% of the fathers are between the ages of 25–35, 60.6% are between the ages of 36–45, and 12.8% are 46 years and over. According to education level, 8.9% of fathers are primary school graduates, 25.7% high school graduates, 8.2% associate degree graduates, 44.0% were undergraduate and 13.3% postgraduate graduates. Regarding participation in their children’s development and life, 53.8% of fathers stated that they participate mostly, 25.7% sometimes, and 20.4% always. While 52.6% of fathers spend 1–3 hours a day with their children, 39.2% spend 4 hours or more, and 8.2% spend less than 1 hour a day. Among fathers, 45.0% felt competent, 11.8% very competent, 33.9% somewhat competent, and 9.4% not competent at all in answering questions about their child’s sexual development. While 69.0% of fathers reported that they had not previously received information about children’s sexual development, 31.0% stated that they had.

Of the children, 53.8% were male, 46.2% were female, 56.2% were aged of 7–11, and 43.8% were aged 3–6. Regarding family size, 32.0% of fathers had one child, 51.1% had two children, and 16.9% had three or more children. While 35.1% of fathers have only male children and 27.4% only female children, 37.5% have both male and female children.

Household income was highest in the 20,001–40,000 TL range (27.6%), followed by 80,001 TL and above (23.3%), 40,001–60,000 TL (23.2%), 60,001–80,000 TL (14.1%), and 0–20,000 TL (11.8%).

### Data collection tools

#### Socio-Demographic Information Form.

Information about the participant fathers’ age, marital status, education level, occupation, employment status, number of children, children’s gender, time spent with the child during the day, and sense of competence in in contributing to their children’s sexual development was obtained.

#### Childhood Trauma Questionnaire Short Form (CTQ-SF).

The Childhood Trauma Questionnaire Short Form was developed by Bernstein et al. [54] to assess individuals’ experiences of abuse during childhood. The internal consistency coefficients of the scale were found to be between 0.84–0.89 for emotional abuse, 0.81–0.86 for physical abuse, 0.92–0.95 for sexual abuse, 0.85–0.91 for emotional neglect and 0.61–0.78 for physical neglect across different groups. It has been reported that the CTQ-SF demonstrates sufficient validity and reliability and can be applied to different populations (clinical and non-clinical samples). Both the original and the Turkish form of the CTQ-SF adapted by Kaya [55] consist of 28 items and 5 sub-dimensions. The scale consists of 5 sub-dimensions: Physical abuse (Items 9, 11, 12, 15, and 17), Sexual abuse (Items 20, 21, 23, 24, and 27), Emotional abuse (Items 3, 8, 14, 18, and 25), Physical neglect (Items 1, 2, 4, 6, and 26), and Emotional neglect (Items 5, 7, 13, 19, and 28). The CTQ-SF is a Likert-type measurement tool ranging from ‘1 strongly disagree’ to ‘5 strongly agree’. Items that should be reverse-scored are items 2, 5, 7, 13, 26, and 28. The lowest possible score on the scale is 25, and the highest is 125. The 3 control items (10, 16 ve 22) are not included in the scoring. Higher scores on the scale indicate more severe experiences of childhood abuse. In the Turkish validity and reliability study, the internal consistency Cronbach’s alpha coefficient of the CTQ-SF was calculated as 0.77 for the total score [55]. To gain a clearer understanding of the content and measurement focus of the CTQ-SF, several sample items are presented: “When I was a child, I was punished by being beaten with a belt, stick, cable, or a similar hard object” (Physical abuse), “When I was a child, someone (or multiple people) attempted to touch me for sexual purposes or asked me to touch them” (Sexual abuse) and “When I was a child, I believed that my mother and father wished I had never been born” (Emotional neglect).

#### Social Anxiety Scale Short Form.

Nunes et al. [56] revised the original 18-item version developed by La Greca et al. [57] into a 12-item short form to assess individuals’ social anxiety levels. The scale includes three sub-dimensions: “Fear of negative evaluation (Items 1-4)”, “Social fear and uneasiness in new situations (Items 5-8)”, and “Social fear and uneasiness in general situations (Items 9-12)”. It is a Likert-type scale ranging from ‘1 Strongly disagree’ to ‘5 Strongly agree’. The highest possible score on the scale is 60, and the lowest is 12. High scores on the scale indicate that the individual has high levels of social anxiety. In the reliability analyses, the internal consistency Cronbach’s alpha coefficient was reported as 0.90. The internal consistency Cronbach’s alpha coefficients for the total and sub-dimensions of the scale adapted into Turkish by Can and Bozgün [58] were calculated as 0.78–0.92. As a result of their Confirmatory Factor Analysis (CFA), χ2/sd: 3,205; RMSEA:.072; GFI:.939; CFI:.955; SRMR:.044 indicated that the structural model fit index values of the scale were at an acceptable level [58]. To gain a clearer understanding of the content and measurement focus of the Social Anxiety Scale, several sample items are presented: “I worry that others will not like me.” (Fear of Negative Evaluation), “I feel uneasy when I meet new people.” (Social Fear and Discomfort in New Situations) and “I hesitate to suggest doing something with others (e.g., going to the cinema) because they might say no.” (Social Fear and Discomfort in General Situations).

#### Rosenberg Self-Esteem Scale.

The Rosenberg Self-Esteem Scale was developed by Morris Rosenberg (1965) to assess individuals’ levels of self-esteem. Its adaptation to Turkish culture was carried out by Çuhadaroğlu [59]. The scale consists of 10 Items, comprising five positive (Items 1, 2, 4, 6, and 7) and five negative (Items 3, 5, 8, 9, and 10) statements, and is a Likert-type scale ranging from “a) Very True” to “d) Very False”. The questions are scored by Guttman evaluation method. Scoring is performed using the Guttman evaluation method. Specifically, Items 1–3 are evaluated together, as are Items 4 and 5 and Items 9 and 10. If the respondent endorses any two of the first three items, they receive one point for that set. Similarly, endorsing a scoring option in either Item 4 or Item 5, or in either Item 9 or Item 10, results in one point for each respective set. Items 6, 7, and 8 are scored individually. Thus, respondents who endorse all applicable items may obtain a maximum total score of 6 points.

Self-esteem levels are classified as follows: 0–1 point indicates high self-esteem, 2–4 points indicate moderate self-esteem, and 5–6 points indicate low self-esteem. The Cronbach’s alpha reliability coefficient reported in the Turkish adaptation study was.76, and the test–retest reliability coefficient calculated at four-week intervals was 0.71. To gain a clearer understanding of the content and measurement focus of the Rosenberg Self-Esteem Scale, several sample items are presented: “I consider myself at least as valuable as other people.” and “I generally tend to see myself as an unsuccessful person.”

#### Attitude Scale Towards Sexual Education (ASSE).

The Sexual Education Attitude Scale was developed by Ceylan, Artan and Kurnaz Adıbatmaz [60] to assess parents’ attitudes towards sexual education. The scale consists of 39 items and four sub-dimensions: Professional support and respect for diversity (Items 1–8), Role of parent (Items 9–16), Belief in its necessity (Items 17–28) and Avoidance (Items 29–39). It is a 4-point Likert-type scale ranging from “1 – Strongly Disagree” to “4 – Strongly Agree.” The items in the Avoidance sub-dimension are reverse-scored. The lowest possible score on the scale is 39, and the highest is 156. A higher score indicates a positive attitude towards sex education. The Cronbach’s alpha for the sub-dimensions and the total scale score range from 0.85 to 0.93. To gain a clearer understanding of the content and measurement focus of the ASSE, several sample items are presented: “I would like to receive support from experts (professionals or guidance counselors) during the process of providing sexual education to my child.” (Professional support and respect for diversity), “I create opportunities for my child to ask questions about sexuality.” (Role of parent), “My child asking questions about sexuality makes me uncomfortable.” (Avoidance).

#### Sexual Communication Scale for Parents (SCSP).

The Sexual Communication Scale for Parents was developed by Artan, Ceylan, and Kurnaz Adıbatmaz [45] to assess the communication language used by parents during sexual education with their children. The scale consists of 23 items and four sub-dimensions: Content (Items 1–7), Body language (Items 8–12), Process management (Items 13–19), and Anxiety (Items 20–23). Only the anxiety sub-dimension is reverse-scored. It is a 4-point Likert-type scale ranging from “1 – Not suitable for me at all” to “4 – Completely suitable for me.” The lowest possible score on the scale is 23, and the highest is 92. A higher score on the scale indicates that the language parents use during sexual education is more consistent with an appropriate sexual communication language. The internal consistency Cronbach’s alpha coefficient for the sub- dimensions and the total scale were reported to range between 0.70 and 0.80 based on analyses conducted with two different sample groups, indicating acceptable reliability. To gain a clearer understanding of the content and measurement focus of the SCSP, several sample items are presented: “I explain, in an age-appropriate way, that others cannot touch their body without limits” (Content), “I speak with my usual facial expression” (Body language), “I try to control my excitement” (Process management) and “Even if I know their answer, I change the topic” (Anxiety).

### Data analysis

The data for this study were analyzed using the open-source analysis program R and SPSS v24 statistical software. The main R packages used included “lavaan,” “semPlot,” “readr,” and “dplyr.” Before starting the analyses, the characteristics of the observed scales were examined and the assumptions of the analyses were checked. The normality assumption was evaluated using skewness and kurtosis coefficients. The decision criterion varies depending on the sample size. For samples with n > 300, if the absolute skewness value exceeds ± 2.0 and the kurtosis value exceeds 7.0, it is decided that the data are not normally distributed [61]. The internal consistency of the scales was determined using Cronbach’s Alpha Coefficient (α), which is one of the most commonly used methods for Likert-type scales [62].

Additionally, the data were screened for multicollinearity using the Variance Inflation Factor (VIF), and variables with VIF values greater than 5 were excluded from further analyses to ensure the independence of predictors. A comprehensive assessment of the validity and reliability of the scales used in the study was subsequently conducted. Because SEM requires the measurement model demonstrate adequate fit before testing the structural relationships, a series of confirmatory factor analyses (CFAs) were conducted to ensure that each latent construct was well defined and psychometrically sound. CFA was performed using various fit index values, including CFI, TLI, SRMR, and RMSEA. To be considered acceptable, the fit index values should meet the following criteria: CFI > 0.90, TLI > 0.90, SRMR < 0.08, and RMSEA < 0.08 [47,48,63].

Each measurement model (childhood trauma, social anxiety, attitudes toward sexual education, and father-child sexual communication) was tested separately. Items with standardized factor loadings below 0.40 were considered for removal; however, removal decisions were guided by both empirical and theoretical justifications. Specifically, items were excluded only if their low loading indicated poor conceptual consistency or redundancy with other items. During the SEM (Structural Equation Modeling) phase, all factors and their structural relationships were established. The models were tested using maximum likelihood estimation, and fit indices were calculated to evaluate model adequacy. Model refinement was performed iteratively, integrating both statistical indicators (e.g., non-significant paths, modification indices) and theoretical reasoning derived from the literature. Non-significant paths were removed only when they lacked conceptual support or contradicted theoretical expectations consistent with previous findings on the psychological mechanisms linking childhood adversity, self-esteem, and communication outcomes [49,64]. Similarly, additional covariances were added to the model only when they represented theoretically plausible associations (e.g., conceptually related dimensions of similar constructs). Each added covariance was evaluated in terms of both empirical necessity and theoretical coherence to avoid overfitting and to enhance model interpretability.

The final SEM model demonstrated satisfactory fit (CFI > .90, TLI > .90, RMSEA < .08, SRMR < .05), suggesting that the hypothesized structure was adequately supported by the data. Relationships between measurements were examined using the Pearson product-moment correlation coefficient. Subsequently, the assumptions for SEM analysis were checked, and an SEM model was constructed to demonstrate the causal relationship between an independent variable (Adverse Childhood Experiences) and a dependent variable (Father-child sexual communication language) through latent variables and observed causes.

Detailed results of the CFA, including fit indices, removed items, and theoretical justifications, are presented in S1 and S2 Tables. Furthermore, a transparent summary of the model refinement process including non-significant path removals, added covariances, and fit improvements across model iterations is provided in S3 and S5 Tables.

### Findings

In this section of the study, the analysis of the data obtained is presented.

When Table 2 is examined, it is seen that childhood trauma have a statistically significant negative relationship with self-esteem (r = −.405, p < 0.01), a positive relationship with social anxiety (r = .331, p < 0.01) and a negative relationship with sexual communication (r = −.229, p < 0.01). However, even if there is a negative relationship with attitudes towards sexual education (r = −.076, p > 0.05), this does not express a statistically significant difference. Self-esteem has a negative relationship with social anxiety (r = −.405, p < 0.01) but a statistically significant positive relationship with attitudes towards sexual education (r = .132, p < 0.01) and sexual communication (r = .279, p < 0.01). Social anxiety showed a statistically significant negative relationship with attitudes towards sexual education (r = −.282, p < 0.01) and sexual communication (r = −.431, p < 0.01). Attitudes towards sexual education showed a statistically significant positive relationship with sexual communication (r = .538, p < 0.01). Attitudes towards sexual education show the highest relationship with sexual communication. When Table 2 is analysed, it is seen that there is no multicollinearity (r > 0.90) [63].

**Table 2:** Correlations and descriptive statistics of variables.

Variables	Correlation Coefficients (r)					Descriptive Statistics		
	1	2	3	4	5	M	Ss	α
1. Childhood trauma	1	−.405^**^	.331^**^	−.076	−.229^**^	40.86	10.17	0.87
2. Self-esteem		1	−.405^**^	.132^**^	.279^**^	1.09	1.21	0.64
3. Social anxiety			1	−.282^**^	−.431^**^	24.38	9.75	0.93
4. Attitude towards sexual education				1	.538^**^	125.51	18.21	0.95
5. Sexual communication					1	80.86	8.95	0.91

**Correlation is significant at the 0.01 level (2-tailed). *Correlation is significant at the 0.05 level (2-tailed).

Although the reliability coefficient for the Self-Esteem Scale was marginal (α = 0.64), it was retained in the model due to its theoretical relevance, acceptable psychometric use in previous studies, and adequate sample size supporting SEM assumptions.

### Analysis results of the research model

**Fig 1:**
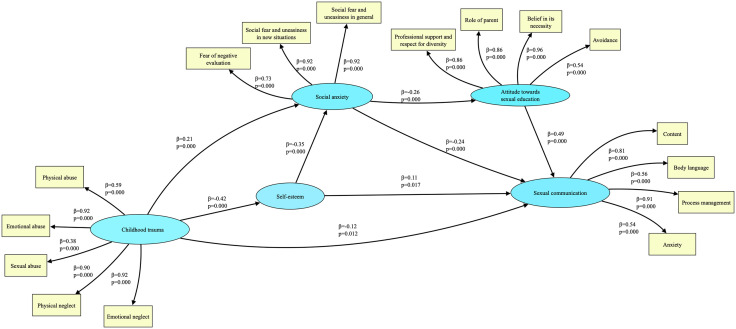
The conceptual framework of the study and the mediating role of self-esteem, social anxiety and attitudes towards sexual education in the relationship between childhood trauma and sexual communication.

It was observed that the fit indices (χ2 = 4442.274, df = 2314, RMSEA = 0.040, SRMR = 0.057, CFI = 0.914, TLI = 0.911, AIC = 72965.946, BIC = 73714.075) of the model created within the scope of the study (Fig 1) showed a good fit.

When Table 3 is examined, the study examines the associations between childhood trauma, self-esteem, social anxiety, attitudes towards sexual education, and father–child sexual communication language. Additionally, the relationships between these variables were evaluated. Childhood trauma was negatively and significantly associated with self-esteem (β = −0.42, p < .001) and sexual communication (β = −0.12, p = .012), and positively associated with social anxiety (β = 0.21, p < .001). It was observed that self-esteem was negatively associated with social anxiety (β=−0.35, p < .001) and positively associated with sexual communication (β = 0.11, p = .017). When examining the associations involving social anxiety, it showed the strongest negative association with attitudes towards sexual education (β=−0.26, p < .001) and sexual communication (β=−0.24, p < .001). Attitudes towards sexual education were observed to be a variable with a strong positive relationship with sexual communication (β = 0.49, p < .001). These findings indicate that the father-child sexual communication language is strongly associated with social and psychological factors.

**Table 3:** Standardized direct effects of self-esteem, social anxiety and attitudes towards sexual education on the relationship between childhood trauma and sexual communication.

LHS	RHS	β	p
Childhood trauma	Self-esteem	−0.42	<.001
Childhood trauma	Social anxiety	0.21	<.001
Childhood trauma	Sexual communication	−0.12	.012
Self-esteem	Social anxiety	−0.35	<.001
Self-esteem	Sexual communication	0.11	.017
Social anxiety	Attitudes towards sexual education	−0.26	<.001
Social anxiety	Sexual communication	−0.24	<.001
Attitudes towards sexual education	Sexual communication	0.49	<.001

Childhood trauma (β=−0.00, p = .967) and self-esteem (β=−0.01, p = .787) were not found to have a significant relationship on attitudes towards sexual education. These pathways were removed from the model.

Upon examining Table 4, it is observed that although the effect of childhood trauma on social anxiety via self-esteem has decreased, the indirect effect remains statistically significant and positive (β = 0.144, p < .001, 95% CI [0.101, 0.187]). This finding indicates that self-esteem plays a mediating role in fathers experiencing social anxiety processes. It has been observed that childhood trauma is negatively associated with attitudes towards sex education through social anxiety (β=−0.056, p < .001, 95% CI [−0.087, −0.026]). This finding demonstrates that social anxiety mediates the development of negative attitudes toward sexual education among fathers. Although the association between childhood trauma and fathers’ sexual communication language through self-esteem decreases, the statistically significant negative effect persists (β=−0.044, p = .023, 95% CI [−0.064, −0.024]). This finding indicates that self-esteem mediates the use of positive sexual communication language among fathers. Similarly, it is observed that childhood trauma negatively associated with sexual communication through social anxiety (β=−0.050, p = .002, 95% CI [−0.079, −0.021]). This finding also highlights the mediating role of social anxiety in fathers’ use of sexual communication language.

**Table 4:** The standardized indirect effects of self-esteem, social anxiety, and attitudes toward sexual education on the relationship between childhood trauma and sexual communication.

Indirect effect	β (Estimate)	p	95% LLCI	95% ULCI
Childhood trauma → Self-esteem → Social anxiety	0.144	0.000	0.101	0.187
Childhood trauma → Social anxiety → Attitudes towards sexual education	−0.056	0.000	−0.087	−0.026
Childhood trauma → Self-esteem → Sexual communication	−0.044	0.023	−0.064	−0.024
Childhood trauma → Social anxiety → Sexual communication	−0.050	0.002	−0.079	−0.021
Childhood trauma → Self-esteem → Social anxiety → Attitudes towards sexual education	−0.038	0.000	−0.055	−0.021
Childhood trauma → Self-esteem → Social anxiety → Sexual communication	−0.034	0.000	−0.051	−0.017
Childhood trauma → Social anxiety → Attitudes towards sexual education→ Sexual communication	−0.027	0.000	−0.043	−0.012
Childhood trauma → Self-esteem → Social anxiety → Attitudes towards sexual education → Sexual communication	−0.018	0.000	−0.027	−0.010

It was also found that childhood trauma had a negative indirect associated with attitudes toward sexual education through self-esteem and social anxiety (β=−0.038, p < .001, 95% CI [−0.055, −0.021]). Similarly, childhood trauma also had a negative indirect effect on sexual communication through self-esteem and social anxiety (β=−0.034, p < .001, 95% CI [−0.051, −0.017]). This sequential pattern indicates that lower self-esteem is linked with higher social anxiety, which in turn is associated with more negative attitudes toward sexual education and less positive sexual communication.

It was further observed that childhood trauma, without accounting for self-esteem, had a significant indirect effect on sexual communication through social anxiety and attitudes toward sexual education (β=−0.027, p < .001, 95% CI [−0.043, −0.012]). This suggests that higher social anxiety is associated with more negative attitudes toward sexual education, which in turn is associated with weaker father–child sexual communication. Childhood trauma demonstrated a multi-step negative effect on sexual communication through self-esteem, social anxiety, and attitudes toward sexual education (β=−0.018, p < .001, 95% CI [−0.027, −0.010]). This pathway represents the longest sequential pattern, in which the negative relationship between childhood trauma and low self-esteem is accompanied by higher social anxiety and more negative attitudes toward sex education, which are in turn associated with less positive sexual communication.

The pathways “Childhood trauma → Self-esteem → Attitudes toward sexual education” and consequently to “Sexual communication” were found to be statistically insignificant (p = 0.787) and were removed from the model. These findings suggest that attitudes toward sexual education are more strongly associated with social anxiety than with self-esteem alone.

## Discussion

This study provides insights into the sexual communication language fathers use when talking to their children aged 3–11, in relation to their childhood trauma experiences. To examine the dynamics underlying these associations, a detailed path analysis was conducted, considering the potential mediating roles of fathers’ self-esteem, levels of social anxiety, and attitudes toward sexual education in the relationship between their childhood trauma experiences and sexual communication language. Although the central role of parenting in children’s healthy growth and development has been strongly emphasized [7,8,65], the involvement of fathers in their children’s sexual development remains understudied and warrants further investigation. Given the importance of early and accurate sexual education and communication for individuals’ well-being, success, and relationships with others, this study focused on fathers of children aged 3–11. We believe our study provides valuable insights into the factors associated with fathers’ sexual communication language.

Our findings, consistent with the literature, indicate that the sexual communication language fathers establish use with their children is associated with their childhood trauma experiences, self-esteem, social anxiety, and attitudes toward sexual education. According to these findings, as fathers’ childhood trauma experiences and levels of social anxiety decrease, and as their self-esteem and positive attitudes toward sexual education increase, the accuracy and quality of the sexual communication language they use with their children also improve. Additionally, the study highlights the multifaceted and significant associations between childhood trauma experiences and critical psychosocial factors in adulthood, such as self-esteem, social anxiety, and father-child sexual communication. In particular, when examining the indirect effects, sequential pathways such as *childhood trauma experiences → self-esteem → social anxiety → attitudes toward sexual education/sexual communication language*, should be interpreted as patterns of association that are consistent with theoretical expectations rather than causal pathways. Nonetheless, these patterns emphasize the need for multilayered interventions targeting mental health, attitudes toward sexual education, and sexual communication language. Furthermore, it was observed that positive attitudes toward sexual education strongly correlate with father’s sexual communication language. These findings underscore the importance of interventions for fathers who have experienced childhood trauma, emphasizing not only trauma-focused approaches but also addressing issues of self-esteem and social anxiety.

When the relevant literature is reviewed, it is observed that, similar to our study, childhood trauma experiences are associated with self-esteem [34–37], social anxiety [30–33], as well as close relationships and communication [38,39,66]. Self-esteem is also found to be associated to anxiety [30,31,67] and communication [68–70]. Similarly, social anxiety is noted to be associated with communication [71,72]. Additionally, it has been stated that social anxiety is related to parents’ attitudes toward sexual education as parents with higher levels of social anxiety may fear being judged as “inappropriate” when providing sexual information to their children [3]. Moreover, many studies have shown that attitudes toward sexual education are closely associated with sexual communication language [15,17,18,60,73,74]. Consistent with previous studies, the present study identified the mediating roles of self-esteem, social anxiety, and attitudes toward sexual education in relation to fathers’ sexual communication language.

According to the findings of our study, childhood trauma experiences show both direct and indirect associations with the sexual communication language that fathers use with their children. The direct association appears in the negative relationship between childhood trauma experiences and sexual communication language. The indirect associations, on the other hand, emerge through variations in fathers’ self-esteem and social anxiety. Considering that parents are the primary sexual educators of their children [75], it is reasonable to assume that the appropriate use of sexual communication language is significantly associated with parents’ childhood trauma histories, mental health, and attitudes toward sexual education [6,15,17,18,76]. The indirect associations identified in our study highlight the critical role of self-esteem, social anxiety, and attitudes toward sexual education in the relationship between childhood trauma experiences and parental sexual communication language. Our results suggest that childhood trauma experiences are linked to less effective sexual communication language among fathers, potentially through lower self-esteem and higher social anxiety, which in turn are related to more negative attitudes toward sexual education. Interestingly, the path from self-esteem to attitudes toward sexual education was not statistically significant in our model. This non-significant relationship suggests that self-esteem, although central to general self-evaluation, may not directly influence how fathers cognitively or emotionally appraise sexual education. In contrast, social anxiety appears to have a stronger association with attitudes toward sexual education, likely because it reflects fears of judgment, embarrassment, or perceived social disapproval factors that are especially salient in conversations about sexuality. In culturally conservative societies such as Turkey, fathers may feel constrained by gender norms that define sexual communication as inappropriate or the mother’s responsibility. As a result, even fathers with relatively high self-esteem may avoid expressing positive attitudes toward sexual education due to anxiety about violating cultural expectations or being judged by others. This interpretation aligns with evidence showing that fear of social evaluation often outweighs self-perception socially sensitive contexts. Previous research has shown that individuals who have experienced childhood abuse often have low self-esteem and are more likely to report negative parenting behaviors [35,55,77]. Experiencing three or more adverse childhood experiences has been associated with social anxiety in adulthood [78], and it has been noted that such traumas can be linked to disruptions in well-being, lower self-esteem, and greater social isolation [79]. The relationship between bullying, self-esteem, and social anxiety further demonstrates the mediating role of self-esteem in social anxiety [80]. Additionally, studies have revealed a negative relationship between effective and healthy communication skills and social anxiety [81] and a positive relationship between communication skills and self-esteem [82]. These findings align with the literature, demonstrating that childhood trauma experiences are associated with lower levels of fathers’ self-esteem, higher levels of social anxiety, and differences in communication skills through these related psychosocial variables.

As seen in Fig 1, emotional abuse, emotional neglect, and physical neglect exhibit the strongest associations among the latent indicators of childhood trauma in the model. Previous research indicates that individuals who were exposed to emotional abuse and neglect in childhood tend to report higher levels of social anxiety than those who were not exposed [83,84]. The prominence of emotional abuse and neglect among the latent indicators of childhood trauma reinforces the interpretation that relational and emotional deprivation, rather than strictly physical harm may exert a more enduring effect on fathers’ psychosocial functioning. These early experiences are closely linked to reduced self-esteem and heightened vulnerability to social anxiety, the two central mediators in our model. The strong loadings of emotional abuse and neglect variables provide additional empirical support for the associational pathway suggesting that disrupted early emotional experiences may affect fathers’ confidence and anxiety in social and parenting contexts, ultimately influencing the language they use when communicating about sexuality with their children. The latent indicators of social anxiety in our model are consistent with these findings. Regarding fathers’ attitudes toward sexual education, the weakest association appears in the latent indicator of avoidance, whereas stronger associations are observed in believing in its necessity, recognizing the parental role, valuing professional support, and respecting differences. Prior studies show that although most parents believe it is important to talk about sexuality with their pre-adolescent children, they struggle to put these conversations into practice [5,85,86]. This shows that parents are aware of their role and believe in the necessity of sexual education, but they cannot fulfill this role sufficiently [85,87,88]. Similarly, the associations observed in the latent indicators of content and process management within sexual communication language align with the underlying reasons for fathers’ attitudes toward sexual education. Many studies report that parents are mostly concerned about when to start, what information to provide, and how to explain it [15,17,18,73,74]. In this context, our findings indicate that fathers’ sexual communication language with their children is shaped by their childhood experiences, psychological processes, and the attitudes they adopt. The current study contributes to the literature by identifying key factors that influence both positive and negative communication attempts of fathers in the process of sexual communication language with their children.

### Limitations, research recommendations and implications

There are some limitations to the present research. Although Turkey is a country where people of many ethnic origins (Syria, Iraq, etc.) live, this study included only literate Turkish citizen fathers who shared the same living space with their children. The main reason for this limitation is the importance of fathers having opportunities to communicate and the fact that the scales used in the study were adapted to Turkish or developed for individuals in Turkey. Future studies could adapt these scales for use with different ethnic groups and test the model with fathers from diverse backgrounds. Such culturally specific research would enrich the literature on fathers’ attitudes toward sexual education and the sexual communication language they use with their children. Additionally, further research is needed to reflect the potential influences and associations of variables related to social norms and cultural values. Our sample included fathers who voluntarily participated and had children aged 3–11 years; therefore, more studies are required to improve the generalizability of our findings. Another limitation concerns the internal consistency of the Self-Esteem Scale, which showed a Cronbach’s alpha 0.64 in this study. This marginal reliability level may have weakened slightly attenuated the structural paths in the SEM analysis. Future research could employ alternative or revised versions of the scale with higher reliability to strengthen measurement validity and model robustness. Another limitation concerns the modeling of childhood trauma. In this study, multiple forms of childhood trauma were represented under a single latent construct. While this approach accounts for shared variance and measurement error, individual trauma types particularly sexual trauma may exert distinct effects on fathers’ attitudes toward sexual education. Despite these limitations, our study makes an important contribution by highlighting the role of self-esteem, social anxiety, and attitudes toward sexual education in understanding how fathers’ childhood abuse experiences relate to the sexual communication language they use with their children aged 3–11 years. Moreover, given that most existing studies focus solely on mothers, limiting generalizations about fathers, this research addresses a significant gap in the literatüre. These findings are useful for professionals working on sexual development and sexual health, as they provide information about possible factors associated with the language of sexual communication fathers use with their children. Many children and adolescents encounter contradictory, negative and confusing messages about sexuality, and in many societies, prevailing attitudes, laws, and social norms limit or discourage open public discussion of sexual topics [1]. Such negative communication patterns can lead to restricted or inadequate knowledge. Individuals who experience limited family communication about sexuality or who feel anxious about sexual topics are more likely to continue this pattern into adulthood and may avoid discussing sexuality with their own children. This continuation occurs because many parents replicate the sexual communication patterns they learned from their own parents [3,6]. Enhancing parental competence has been shown to relate to both the effectiveness and frequency of sexuality-related conversations [17,41]. Experts can identify the factors that relate to father-child sexual communication language and organize psycho-education programs that will enable fathers to take an active role in children’s sexual development. Facilitating open and positive sexual communication with parents may be linked to the child’s self-confidence, adaptation to society and healthy communication with the environment.

Beyond individual and familial factors, these findings should also be interpreted within broader cultural and policy frameworks. In Turkey, where sexuality is often regarded as a private or even taboo topic, fathers may feel additional pressure to avoid such conversations, highlighting the need for culturally sensitive approaches that normalize parent–child dialogue about sexual issues. Integrating evidence-based parenting and communication modules into existing school curricula, community health programs, and father-focused interventions may help promote healthy sexual communication within families. Collaboration among schools, public health institutions, and community-based organizations could provide structured opportunities for fathers to develop communication confidence and culturally sensitive skills for discussing sexual topics with their children. Moreover, national policies that support family and sexuality education could play a transformative role in fostering open dialogue, reducing stigma, and promoting children’s sexual well-being.

Trauma-informed education sessions can help fathers recognize how early adverse experiences may relate to emotional regulation and parenting, while promoting empathy and self-awareness. These programs may incorporate self-esteem enhancement components, such as reflective exercises and peer feedback activities, to support fathers in developing a more positive sense of self and parenting competence. Given the strong mediating role of social anxiety, interventions should also incorporate strategies to reduce fear of social judgment such as group-based activities, guided discussions, and cognitive-behavioral techniques that enhance fathers’ confidence in communicating about sensitive issues. Targeted training in communication skills can further emphasize developing open, age-appropriate, and value-consistent dialogue with children. Embedding these components into community and school-based programs through interdisciplinary collaboration would ensure that fathers receive both emotional support and practical skills to engaging in constructive sexual communication with their children. Future research is also recommended to model different types of childhood trauma as separate latent or observed variables, which would allow for a more precise examination of the unique contributions of each trauma type makes to fathers’ sexual communication attitudes and behaviors.

## Supporting information

S1 Table CFA fit indices by latent construct. (DOCX)

S2 Table Items removed during CFA and empirical/theoretical justification. (DOCX)

S3 Table Structural paths removed during SEM model refinement. (DOCX)

S4 Table Added covariances during SEM refinement and justification. (DOCX)

S5 Table Model refinement process and comparative fit indices. (DOCX)
