# Supplementary material for: The role of childhood traumas on father-child sexual communication language: Self-esteem, social anxiety and sexual education
Source: PLoS One. 2026 Mar 5;21(3):e0340776. doi: 10.1371/journal.pone.0340776 (PMC12962492; doi:10.1371/journal.pone.0340776)
Supplement: S1 Table — (DOCX) [file pone.0340776.s001.docx]

**S1 Table.**

CFA Fit Indices by Latent Construct

| **Scale / Factor** | **χ²/df** | **CFI** | **TLI** | **RMSEA** | **SRMR** | **Evaluation** |
| --- | --- | --- | --- | --- | --- | --- |
| **Childhood Trauma** | 3.67 | .948 | .935 | .067 | .049 | Acceptable fit |
| **Social Anxiety** | 3.43 | .978 | .970 | .064 | .029 | Acceptable fit |
| **Attitudes Toward Sexual Education** | 4.68 | .893 | .880 | .079 | .050 | Acceptable fit |
| **Father–Child Sexual Communication** | 4.09 | .928 | .913 | .073 | .052 | Acceptable fit |

Note. Acceptable model fit was determined using the following cutoffs: CFI/TLI ≥ .90, RMSEA ≤ .08, and SRMR ≤ .08. Each measurement model was tested separately prior to the structural phase.
