# Supplementary material for: The role of childhood traumas on father-child sexual communication language: Self-esteem, social anxiety and sexual education
Source: PLoS One. 2026 Mar 5;21(3):e0340776. doi: 10.1371/journal.pone.0340776 (PMC12962492; doi:10.1371/journal.pone.0340776)
Supplement: S2 Table — (DOCX) [file pone.0340776.s002.docx]

**S2 Table.**

Some sample items for Items Removed During CFA and Their Empirical/Theoretical Justifications

| **Scale** | **Item** | **Std. Lv** | **Decision** | **Justification (Empirical & Theoretical)** |
| --- | --- | --- | --- | --- |
| **Childhood Trauma** | Item 17 | .31 | Removed | Low loading, inconsistent with “Physical Abuse” domain |
|  | Item 4 | .12 | Removed | Weak loading, poor conceptual coherence with “Physical Neglect” |
|  | Item 19 | -73 | Removed | Inconsistent with subscale “Emotional Neglect” |
| **Social Anxiety** | Item 3 | .89 | Removed | high VIF (>5) indicating redundancy |
| **Attitudes Toward Sexual Education** | Item 4 | .54 | Removed | Redundant with similar attitudinal item; conceptually overlapping |
| **Father–Child Communication** | Item 6 | .26 | Removed | Low loading, item did not represent the construct well |

Note. Items were excluded only when both empirical and theoretical criteria justified removal to preserve conceptual integrity.
