# Supplementary material for: The role of childhood traumas on father-child sexual communication language: Self-esteem, social anxiety and sexual education
Source: PLoS One. 2026 Mar 5;21(3):e0340776. doi: 10.1371/journal.pone.0340776 (PMC12962492; doi:10.1371/journal.pone.0340776)
Supplement: S3 Table — (DOCX) [file pone.0340776.s003.docx]

**S3 Table.**

Structural Paths Removed During SEM Model Refinement

| **Path** | **β (Initial)** | **p** | **Decision** | **β (Final)** | **Theoretical Rationale** |
| --- | --- | --- | --- | --- | --- |
| Childhood Trauma → Attitudes Toward Sexual Education | –.03 | .967 | Removed | – | Non-significant; unsupported by prior findings |
| Total Self-Esteem → Attitudes Toward Sexual Education | .04 | .680 | Removed | – | Statistically insignificant, conceptually weak |
