# Supplementary material for: The role of childhood traumas on father-child sexual communication language: Self-esteem, social anxiety and sexual education
Source: PLoS One. 2026 Mar 5;21(3):e0340776. doi: 10.1371/journal.pone.0340776 (PMC12962492; doi:10.1371/journal.pone.0340776)
Supplement: S4 Table — (DOCX) [file pone.0340776.s004.docx]

**S4 Table.**

Added Covariances During SEM Refinement and Theoretical Rationale

| **Residual Covariance** | **MI** | **Standardized Residual Covariance** | **Justification** |
| --- | --- | --- | --- |
| ASSE11 ~~ ASSE 12 | 266.69 | 0.642 | Overlap due to shared content in measurement context |
| ASSE 26 ~~ ASSE 27 | 141.19 | 0.494 | Measures related aspects of the same construct |
| CTQ-SF 28 ~~ CTQ-SF 26 | 55.58 | 0.402 | Potential overlap between physical neglect indicators |
| CTQ-SF 12 ~~ CTQ-SF 14 | 52.88 | 0.340 | Overlap in the context of emotional and physical abuse |
| SCSP 20 ~~ SCSP 21 | 52.78 | 0.338 | Similar items measuring anxiety and concern |
| SCSP 14 ~~ SCSP 15 | 50.75 | 0.334 | Shared content related to management processes |

Note. Covariances were introduced only when both statistical indicators and conceptual rationale supported their inclusion.
