# Supplementary material for: The role of childhood traumas on father-child sexual communication language: Self-esteem, social anxiety and sexual education
Source: PLoS One. 2026 Mar 5;21(3):e0340776. doi: 10.1371/journal.pone.0340776 (PMC12962492; doi:10.1371/journal.pone.0340776)
Supplement: S5 Table — (DOCX) [file pone.0340776.s005.docx]

**S5 Table.**

Model Refinement Process and Fit Index Comparisons

| **Model** | **χ²/df** | **CFI** | **TLI** | **SRMR** | **Notes** |
| --- | --- | --- | --- | --- | --- |
| Initial SEM (without indirect effects) | 2.18 | 0.890 | 0.885 | 0.059 | RMSEA and SRMR good; CFI/TLI borderline acceptable |
| Refined SEM (added residual covariances) | 1.92 | 0.914 | 0.911 | 0.057 | CFI/TLI ≥ 0.90; RMSEA excellent; model fit notably improved |
| Final SEM (non-significant paths removed) | 1.92 | 0.914 | 0.911 | 0.057 | Model simplified; fit indices stable; good fit |

*Note.* Improvements across successive models indicate enhanced parsimony and theoretical coherence rather than mere statistical optimization.
